# Motivators and Barriers to Joining a Lifestyle Change Program for Disease Prevention

**DOI:** 10.1089/whr.2024.0093

**Published:** 2024-09-06

**Authors:** Mandy L. Pershing, Lingzi Zhong, Anthony Ariotti, Kaitlyn Dwenger, Maddie McCarty, Phoebe Freer, Elissa M. Ozanne

**Affiliations:** ^1^ Department of Population Health Sciences, School of Medicine, University of Utah, Salt Lake City, Utah, USA.; ^2^ Department of Communication Sciences and Disorders, College of Health, University of Utah, Salt Lake City, Utah, USA.; ^3^ Division of Otolaryngology, Head and Neck Surgery, School of Medicine, University of Utah, Salt Lake City, Utah, USA.; ^4^ Department of Radiology, School of Medicine, University of Utah, Salt Lake City, Utah, USA.

**Keywords:** lifestyle change programs, weight loss, motivators, barriers, family history of diabetes, ethnicity

## Abstract

**Introduction:**

Lifestyle change programs (LCPs) are effective in helping people adopt healthy lifestyles and maintain healthy weight for disease prevention. LCPs are known to be underutilized, but the nuances surrounding women’s interest in using these programs for disease prevention need to be further explored so that enrollment and retention in these programs can be improved.

**Methods:**

The purpose of this study was to explore women’s interest in and knowledge of LCPs and identify their motivators and barriers to joining these types of programs through a survey. The survey was administered both online and in person. The survey had 22 questions and included demographics, medical and family history, knowledge and interest in LCPs, and barriers and motivators to participating in LCPs.

**Results:**

Participants in this study included 1,606 women from 40 to 74 years of age. We found that respondents had limited knowledge about the benefits of LCPs in reducing risks of specific diseases, such as breast cancer and osteoarthritis. Respondents reported low-to-moderate interest in LCPs. We found that their interest in these programs was negatively associated with their weekly physical activity and positively associated with their body mass index (BMI) and the number of reported barriers to joining LCPs. The most common barriers cited were cost, location, time, and too many meetings. In addition, we found that respondents who had or were unsure about their family history of diabetes were more interested in LCPs compared with individuals who had no family history of diabetes. We did not find significant differences in respondent interest in LCPs across ethnicity.

**Conclusions:**

Our study suggests that specific barriers to LCPs—including women’s knowledge of such programs—will need to be addressed before enrollment and retention in LCPs are increased.

## Introduction

The majority of people living in the United States (71.6%) are either overweight or obese, putting millions of people at risk for diseases such as heart disease, cancer, and diabetes.^1^ While the weight loss industry in the United States is worth an estimated $72 billion,^2^ a combined healthy diet and exercise regimen is the only lifestyle change that shows initial and sustained weight loss.^3–5^ The majority of the US population, however, does not participate in healthy lifestyle choices.^6^ For example, an estimated 13% of the national population (>34 million people) are living with diabetes; of these, >90% of these cases are type 2 diabetes (T2D) and therefore are having a largely preventable condition.^7^ Sustained weight loss through lifestyle changes can alleviate many of the risks for preventable disease in people who are overweight or obese.^8–10^ Hence, there is a substantial need for both educational and motivational support to increase the prevalence of healthy lifestyles in the US population.

Lifestyle change programs (LCPs) are organized efforts with proven methodology to help people adopt healthy lifestyles and reach and maintain a healthy weight. Three evidence-based LCPs with methodologies that proved effective in past clinical trials^11–15^ are the Centers for Disease Control and Prevention’s (CDC) National Diabetes Prevention Program (DPP), Take Off Pounds Sensibly (TOPS), and WeightWatchers (WW). Each of these programs suggests that combining exercise with a healthy diet is more effective for weight loss than either of these programs alone.^16^ Food preferences are an important part of adhering to LCPs, and each of these programs includes education about both diet and exercise. Moreover, education to women in particular is especially important, as women tend to be the primary preparer of food for their families and therefore have influence over the diet of their entire families.

Despite the national need to improve people’s health, evidence-based weight loss programs (*e.g.,* LCPs) remain underused.^17,18^ Up to one-quarter of participants dropped out of major commercial weight loss programs^19^ and approximately one-half of participants did not remain in the National DPP program.^20^ Research has identified several social, practical, and psychological barriers to participant uptake and completion of LCPs, including lack of social support, beliefs, transportation, and costs.^21^ Demographic characteristics—such as ethnicity, age, and gender—are also related to individuals’ enrollment in LCPs.^22^

This research project focused on middle-aged women who are likely to benefit from these lifestyle interventions, as women are more likely than men to be overweight^1^ and women’s weight tends to increase with age.^16,23^ While data suggest there may be common barriers to joining LCPs,^24^ the goals of this research were to (1) further explore women’s awareness of and interest in specific LCPs and (2) identify women’s motivators and barriers to enrollment in LCPs, including specific diseases that would be most motivating to encourage women to lose weight for disease prevention.

## Methods

### Participant recruitment

To increase the representativeness of our study results, we enrolled women from 40 to 74 years of age from multiple settings, resulting in four cohorts: (1) women scheduled for an asymptomatic screening mammogram at a community clinic that serves underserved populations, (2) women scheduled for an asymptomatic screening mammogram at a cancer institute, (3) women who previously expressed interest in being a research participant at the cancer institute, and (4) women recruited through ResearchMatch (https://www.researchmatch.org/about/), a national health volunteer registry created by several academic institutions and supported by the U.S. National Institutes of Health as part of the Clinical Translational Science Award (CTSA) program.^25^ Women who elected to undergo routine screening mammogram are likely to be interested in preventative health and were therefore chosen for our sample. Participants were considered eligible if they were female, were between 40 and 74 years of age, had not been diagnosed with either breast cancer or diabetes, and were not currently pregnant. The survey and research procedures were approved by the University of Utah Institutional Review Board.

### Procedure

Surveys were administered either (1) in person to research participants at the community clinic and at the cancer institute or (2) electronically through ResearchMatch and research participant lists at the cancer institute. Participants who were recruited in person were offered the chance to complete the survey in either English or Spanish. The survey distributed to ResearchMatch participants was in English only.

### Survey instrument

The survey included questions that asked about participant interest in joining an LCP, including three established LCPs (*i.e.,* DPP, TOPs, WW), as well as the amount that they would be willing to pay for an LCP. Participants were also asked to complete questions about their knowledge of the benefits of LCPs, and motivators for and barriers to joining LCPs. Participant interest in joining LCPs was assessed by a five-point Likert scale (0 = not at all interested and 4 = very interested). We also collected demographic information, medical history, and family history of diabetes. Specifically, participants were asked whether they knew if their siblings and parents have been diagnosed with diabetes (yes, type 1 diabetes; yes, type 2 diabetes; and unsure; no).

### Statistical analysis

The R software environment^26^ was used for all statistical analyses. Pairwise deletion was used in analysis with missing data. Analysis of variance (ANOVA) tests were used to examine the association between participant interest in LCPs and family history of diabetes, weekly physical activity, ethnicity, and number of reported barriers. A chi-square test was used to analyze differences in weekly physical activities across ethnicity. Bivariate correlation analyses with Pearson’s *r* were conducted to test the association between BMI and interest in LCPs. Paired-samples t-*test* was used to test differences in participant-reported interest in LCPs that were free versus LCPs with a cost, as well as participant interest in the three established LCPs.

## Results

### Sample characteristics

In total, 1,606 participants who met the eligibility criteria (*i.e.,* age 40–74 years, not diagnosed with breast cancer or diabetes, and not pregnant) completed the survey. See Table 1 for participants’ demographic characteristics. The average BMI of the sample was 28.6, with 63% classified as overweight or obese by CDC standards.^27^ In addition, only 26% of the sample self-reported as meeting the CDC recommendation of 150 minutes of physical activity a week.^28^

**Table 1.** Descriptive Statistics of Study Participants

	Observed frequency	Percentage^a^
Data source	**1,606**	** **
Cancer institute online research platform	99	6.16%
Cancer institute	100	6.23%
Community clinic	54	3.36%
ResearchMatch	1,353	84.25%
Race	**1,585**	** **
American Indian or Alaska Native	10	0.63%
Asian	22	1.39%
Black or African American	72	4.54%
Native Hawaiian and Other Pacific Islander	0	0.00%
White	1,433	90.41%
Multiracial	28	1.77%
Other	20	1.26%
Ethnicity	**1,578**	
Hispanic	55	3.49%
Non-Hispanic	1,392	88.21%
Other	131	8.30%
Weekly physical activity (min)	**1,597**	
None	72	4.51%
30	271	16.97%
30–90	515	32.25%
91–150	328	20.54%
150	411	25.74%
	**Mean**	**SD**
BMI	28.51	10.64

Bold numbers are the total N reported in that category.

^a^ Percentages may not add up to 100% due to rounding.

### Interest in LCPs

**FIG. 1.**
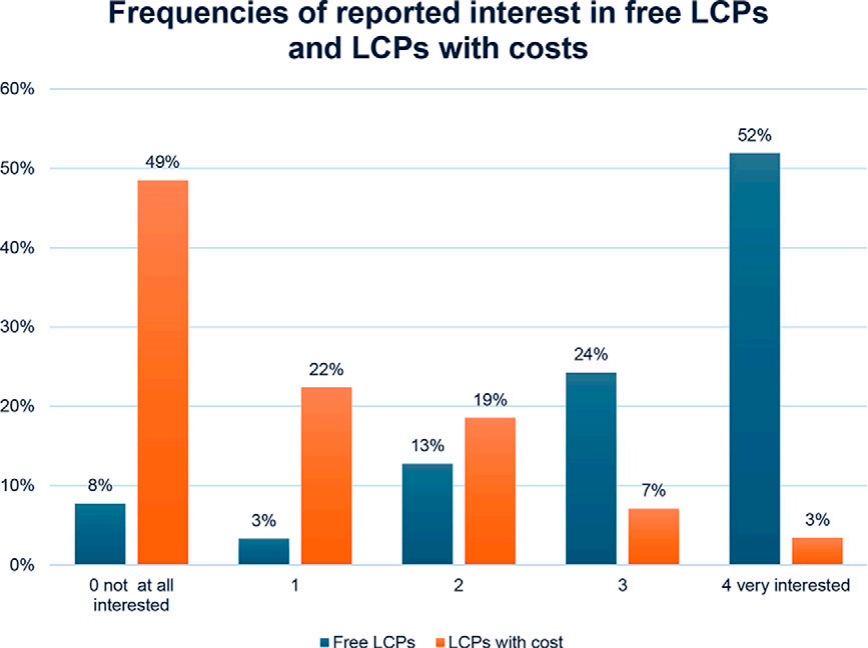
Frequencies of reported interest in free LCPs and LCPs that have a cost. LCP, lifestyle change program.

Overall, participants indicated low to moderate interest in the three established LCPs based on the 0–4 Likert scale (for WW, median = 1; for DPP, median = 2; and for TOPS, median = 2). Out of the three LCP choices, participants indicated significantly greater interest in the DPP compared with WW (*Z* = -.12.08, *p* < 0.001) and TOPS (*Z* = −.9.05, *p* < 0.001). In addition, almost all participants (92.2%) indicated some interest in free LCPs (*i.e.,* indicated interest was >0 on the 0–4 scale), and a majority (76.2%) of participants expressed strong interest (*i.e.,* indicated interest was >2 on the 0–4 scale). The average expressed interest in free LCPs was 3.09 (on the 0–4 scale, SD = 1.21). Slightly more than one-half of the participants (51.5%) expressed some interest in LCPs that had a cost and 10.6% expressed a strong interest in LCPs that had a cost, with an average interest of 0.95 (on the 0–4 scale, SD = 1.12). See Figure 1 for the frequency of expressed interest in LCPs. When asked how much money they would be willing to pay for an LCP overall, the average reported amount was $44.50 (*n* = 1,224, median = $15.00, min = $0, max = $600.00). Spearman’s rank correlation was computed to assess the relationship between age and interest in free LCPs and LCPs with cost. No strong relationship was found between age and LCPs with cost (*r* = 0.02, *p* = 0.391) or free LCPs (*r* = −0.02, *p* = 0.420).

### Knowledge of benefits of LCPs for disease prevention

Participants were asked if they knew that enrolling in an LCP could reduce their risk of diseases, including heart diseases, hypertension, breast cancer, and osteoarthritis. Most (79%) of the participants reported knowing that an LCP could reduce their risk of heart disease and hypertension. Less than half, however, reported knowing that their risk for breast cancer (48%) and osteoarthritis (43%) would be reduced by participating in an LCP.

### Motivators for and barriers to enrolling in LCPs

Several factors were considered as potential motivators for and barriers to participant interest in LCPs, including a variety of diseases, family history of diabetes, BMI, weekly physical activity, and ethnicity. Table 2 outlines the descriptive statistics of participants’ interest in both free LCPs and LCPs that had a cost, as well as the statistical results of the associations among motivators, barriers, and participant interest in LCPs.

**Table 2.** Statistical Analyses Results of the Associations between Interest in LCPs and Family History of Diabetes, BMI, and Numbers of Reported Barriers

	Interest in free LCPs	Interest in LCPs with costs
	**M (SD)**	**M (SD)**
**Sibling diabetes**	**Welch’s *F* = 3.8**	***F* = 5.05** ^**^
Type-1 or type-2 diabetes	3.24 (1.10)	1.07 (1.14)
No	3.06 (1.23)	0.91 (1.11)
Unsure	3.34 (1.06)	1.33 (1.31)
**Parent diabetes**	**Welch’s *F* = 12.3** ^***^	***F* = 8.61** ^***^
Type-1 or type-2 diabetes	3.2 (1.11)	1.13 (1.18)
No	3.03 (1.26)	0.87 (1.09)
Unsure	3.53 (0.74)	1.06 (1.19)
**BMI**	***r* = 0.12** ^***^	***r* = 0.09** ^***^
**Weekly physical activity (mins)**	**Welch’s *F* = 5.7** ^***^	**Welch’s *F* = 4.1** ^**^
None	3.34 (1.05)	0.73 (0.96)
30	3.31 (1.07)	0.96 (1.15)
30–90	3.14 (1.12)	1.04 (1.12)
91–150	3.02 (1.29)	1.03 (1.17)
150	2.92 (1.33)	0.80 (1.09)
**Numbers of reported barriers**	**Welch’s *F* = 16.3** ^***^	**Welch’s *F* = 6.08** ^***^
0	2.10 (1.59)	0.57 (1.05)
1	2.99 (1.30)	0.84 (1.09)
2	3.28 (1.06)	1.05 (1.15)
3	3.24 (1.09)	1.01 (1.13)
4	3.28 (1.04)	0.8 (1.02)
**Ethnicity**	***F* = 0.72**	***F* = 1.14**
Hispanic	2.92 (1.56)	1 (1.17)
Non-Hispanic	3.1 (1.21)	0.93 (1.12)
Other	3.16 (1.03)	1.08 (1.19)

^**^ *p* < 0.01.

^***^ *p* < 0.001.

#### Motivating diseases

Heart disease was the highest motivating disease to join an LCP (*n* = 1,023), with 66% of our sample indicating so, followed by arthritis (*n* = 890, 57.23%), hypertension (*n* = 811, 52.15%), and breast cancer (*n* = 784, 50.42%).

#### Family history of diabetes

Diabetes history for siblings was not found to be significantly associated with participant interest in free LCPs (Welch’s *F* = 3.8, *p* = 0.03^1^), but it was significantly associated with their interest in LCPs that had a cost (*F* = 5.05, *p* = 0.007). Diabetes history for parents was significantly associated with participant interest in free LCPs (Welch’s *F* = 12.3, *p* < 0.001), as well as LCPs that had a cost (*F* = 8.61, *p* < 0.001). Participants who knew that their parent had diabetes were more interested in LCPs that had a cost compared with those who knew their parent did not have diabetes (mean difference = .26, CI = [0.11, 0.4], *p* < .001).

#### BMI

Women with a higher BMI were more interested in both free LCPs (*r* = 0.12, *p* < 0.001, CI = [0.07, 0.18]) and LCPs with costs (*r* = 0.09, *p* < 0.001, 95% CI: [0.04, 0.14]). No difference in BMI was found for different ethnicity groups (*F* = 0.24, *p* = 0.79)^2^.

#### Weekly physical activity

Weekly physical activity was significantly associated with interest in free LCPs (Welch’s *F* = 5.7, *p* < 0.001) and LCPs that had a cost (Welch’s *F* = 4.1, *p* = 0.003). Individuals who reported they got >150 minutes of weekly physical activity were less interested, generally, in LCPs compared with participants who reported fewer hours of weekly physical activity (*e.g.,* 30–90 minutes, <30 minutes). No differences in weekly physical activity were found for different ethnicity groups (*χ^2^* = 13.66, *p* = 0.10).

#### Reported barriers

The most commonly reported barrier was cost (*n* = 1,001), followed by location (*n* = 991), time (*n* = 769), and too many meetings (*n* = 494). Less common barriers were “other” (*n* = 164), too few meetings (*n* = 34), and language (*n* = 24).

Participant interest in free LCPs was significantly different across groups with different numbers of barriers (Welch’s *F* = 16.3, *p* < 0.001), which was also the case for participant interest in LCPs that had a cost (Welch’s *F* = 6.08, *p* < 0.001). Specifically, the more barriers to enrolling in LCPs that participants reported, the more interested they were in LCPs.

## Discussion

There is a national need for an increase in the prevalence of healthy lifestyles and a reduction in disease rates. Women, in their common role as mother and food preparer, have an important role setting expectations for healthy habits in families. Despite the evidence in support of LCPs for weight loss and promotion of healthy lifestyles, as well as their role in preventing disease, there is a paucity in the number of individuals who make use of these types of organized, educational programs.^19^ Therefore, the goal of this study was to explore individuals’—particularly women’s—interest in and knowledge of LCPs and to identify motivating factors and barriers to enrollment in LCPs.

Overall, participants showed only a low-to-moderate amount of interest in participating in LCPs. In addition, participants showed slightly higher interest in the DPP compared with other types of LCPs. Fewer than half of the women surveyed, however, were aware that LCPs could lower their risk for breast cancer and osteoarthritis, but at most, 80% of participants were aware that LCPs could lower their risk for heart disease and hypertension. This study suggests that heart disease and hypertension could be most influential in potentially motivating women to modify their lifestyle to prevent these specific diseases. A previous study that we conducted of both patients and health care professionals who care for women who are at risk for diabetes found that breast cancer, hypertension, and heart disease would be stronger motivators than diabetes for women to lose weight to prevent disease.^4^ Therefore, there is an opportunity for health education on the potential for reducing women’s risk of developing, specifically, breast cancer, osteoarthritis, heart disease, and hypertension through LCP participation, with an end goal of lowering the nation’s disease rate. There is particular opportunity for educating about a healthy lifestyle being important for reducing risk of breast cancer and osteoarthritis, if our survey group is representative of the more general population. It is known that cost, time, and locations are barriers to enrolling and completing an LCP.^24^ It is unknown if knowledge about how an LCP can help prevent these diseases can increase interest in LCPs. Overall, when examining the fact that only one half of this study’s participants knew that the risk of breast cancer and osteoarthritis could be reduced with weight reduction, we can see that there is great opportunity to build on postmenopausal women’s motivation to join an LCP. Therefore, future research on this topic could lend potential for increasing participation in these types of programs.

More participants chose DPP than either WW or TOPS as an LCP that they could potentially participate in. Given that DPP does not seem to be as well-known as the others (WW has advertising directly on grocery store shelves, for example), this was an interesting and unexpected finding. Although both WW and TOPS are older programs, DPP is the only one developed by the CDC, which is a possible indicator of trust and perhaps why there was greater interest shown in that program. At the same time, a previous study^4^ suggested that diabetes prevention was not the greatest motivator to join an LCP. Thus, future studies should examine further the specific characteristics of each program that might potentially motivate women to join to change their lifestyle for disease prevention.

Along with participant interest in and knowledge of LCPs, we also explored motivators for and barriers to participation in LCPs. First, family history of diabetes was significantly associated with interest in LCPs. Participants who had a family history of diabetes or who were unsure of the family history were more interested in LCPs than participants without a family history of diabetes. These findings suggest that individuals who are aware of a family history of diabetes are more vigilant in monitoring their individual health and are thus more motivated to enroll in LCPs. Second, our findings suggested that individuals with greater BMIs and inadequate physical activity were more interested in LCPs, indicating that these individuals have the motivation to change their lifestyle and thus may be prime candidates for LCPs. Taken together, these findings corroborate prior research that has indicated that LCPs should identify individuals who are in the most need of healthy lifestyle changes (*e.g.,* individuals with unhealthy BMIs and family history of chronic illness).^20,29^ It should be noted that despite their interest in LCPs, previous research suggests that individuals with unhealthy BMIs are less likely to remain in LCPs due to barriers such as the demand of exercise.^30^ Furthermore, only about 25% of the sample are getting the CDC-recommended weekly amount of exercise (150 minutes per week), while almost 35% of the sample are classified as being at a healthy weight by BMI standards. These figures are similar to the national averages, where 24.2% of adults in the United States met the CDC-recommended amount of weekly exercise in 2020,^31^ and 30.6% of women are classified as being at a healthy weight.^32^ Hence, more research is needed to identify strategies to retain overweight individuals’ interest in and motivations for changing their lifestyle.

### Limitations

Despite these important findings, this study has some limitations. We did not ask about education, SES, or social support in our survey, which are all factors that impact enrollment and retention in LCPs. We did not assess participant knowledge about different types of diabetes, which could affect how participants responded to the questions about family history of diabetes. In addition, the majority of the participants were non-Hispanic White women, which limit**s** the generalizability of our findings, as it is not a nationally representative sample. However, the range of both age (40–75 years) and BMI (12.2–62.0) in the respondents still provides rich data, even though the data were limited in race and ethnicity diversity. Furthermore, data on food preferences across a diverse population would be helpful for future studies since cultural beliefs about food, including which foods are healthy and which foods are most desirable, vary across a diverse population (such as the United States) that encompasses many food cultures. Finally, we collected only family history of diabetes and did not inquire about participant family history of other diseases (*e.g.,* heart disease, breast cancer, hypertension, osteoarthritis) that are relevant to LCPs and could potentially affect participant interest in LCPs. Thus, future studies endeavoring to learn more about motivators and barriers to joining and maintaining participation in LCPs should collect more detailed family history information and participants’ understanding of family history and diseases, as well as information on participants’ food culture and food preferences.

## Conclusions

This exploratory study shows promise that addressing barriers to joining an LCP and tailoring health education to an individual’s interest in disease risk might increase both enrollment in and maintenance rates of these lifestyle change programs. Greater participation in LCPs could potentially decrease the overall risk of breast cancer, diabetes, hypertension, and osteoarthritis within the population for women 40–74 years of age. The majority of women in our sample were lacking important and possibly motivating information about these programs, such as specific disease risk reduction, indicating the need for increased communication about LCPs from both health organization outreach efforts and possibly from health care professionals themselves. Increased awareness of the health benefits of LCPs may increase women’s interest in joining these programs, especially LCPs that have a cost. Family history may be an important factor in generating interest in LCPs, but more research is needed in this area. By understanding more about motivators for and barriers to weight loss success, future research could focus on developing a randomized controlled trial to test decision aids to educate women older than 40 years and help support their goals for lifestyle change and disease prevention.

## Supplementary Material

Supplementary Appendix S1

## Notes

^1^
Although Welch’s *F* test was significant, Games-Howell post hoc test indicated that there were no significant differences among sibling diabetes history with interests in free LCPs (*p*s > 0.08).

^2^
The results stayed the same when using BMI as a categorical variable (*i.e.*, underweight, healthy weight, overweight, obesity). Participants who were obese and overweight were more interested in LCPs (for free LCPs, Welch’s *F* = 8.75, *p* < 0.001; for LCPs with costs, Welch’s *F* = 3.29, *p* = 0.02) than participants with healthy weight. No difference in BMI was found for different ethnicity groups (*χ^2^* = 3.65, *p* = 0.71).

## Abbreviations Used

ANOVA: Analysis of variance
BMI: Body mass index
CDC: Centers for Disease Control and Prevention
CTSA: Clinical Translational Science Award
DPP: National Diabetes Prevention Program
LCP: Lifestyle change program
T2D: Type 2 diabetes
TOPS: Take Off Pounds Sensibly
WW: WeightWatchers

## References

[1] Centers for Disease Control and Prevention. Prevalence of Obesity and Severe Obesity Among Adults: United States, 2017–2018. 2020.

[2] Business Wire. The $72 Billion Weight Loss & Diet Control Market in the United States, 2019-2023. 2019. Available from: https://www.businesswire.com/news/home/20190225005455/en/The-72-Billion-Weight-Loss-Diet-Control-Market-in-the-United-States-2019-2023–-Why-Meal-Replacements-are-Still-Booming-but-Not-OTC-Diet-Pills–-ResearchAndMarkets.com [Last accessed: January 4, 2022].

[3] Curioni CC, Lourenço PM. Long-term weight loss after diet and exercise: A systematic review. Int J Obes (Lond) 2005;29(10):1168–1174; doi: 10.1038/sj.ijo.0803015 15925949

[4] Miller W, Koceja D, Hamilton E. A meta-analysis of the past 25 years of weight loss research using diet, exercise or diet plus exercise intervention. Int J Obes Relat Metab Disord 1997;21(10):941–947; doi: 10.1038/sj.ijo.0800499 9347414

[5] Volek JS, Vanheest JL, Forsythe CE. Diet and exercise for weight loss. Sports Med 2005;35(1):1–9; doi: 10.2165/00007256-200535010-00001 15651909

[6] Centers for Disease Control and Prevention. Leading Causes of Death. 2022. Available from: https://www.cdc.gov/nchs/fastats/leading-causes-of-death.htm [Last accessed: January 24, 2022].

[7] Centers for Disease Control and Prevention. National Diabetes Statistics Report, 2020. Atlanta, GA; 2020. Available from: https://www.cdc.gov/diabetes/php/data-research/index.html [Last accessed: July 22, 2024].

[8] Hardefeldt PJ, Penninkilampi R, Edirimanne S, et al. Physical activity and weight loss reduce the risk of breast cancer: A meta-analysis of 139 prospective and retrospective studies. Clin Breast Cancer 2018;18(4):e601–e612; doi: 10.1016/j.clbc.2017.10.010 29223719

[9] Chen X, Wang Q, Zhang Y, et al. Physical activity and risk of breast cancer: A meta-analysis of 38 cohort studies in 45 Study reports. Value Health 2019;22(1):104–128; doi: 10.1016/j.jval.2018.06.020 30661625

[10] Freid VM, Bernstein AB, Bush MA. Multiple chronic conditions among adults aged 45 and over: Trends over the past 10 years. NCHS Data Brief 2012(100):1–8. 23101759

[11] Centers for Disease Control and Prevention. National Diabetes Prevention Program—Registry of All Recognized Programs. 2019. Available from: https://www.cdc.gov/diabetes-prevention/programs/what-is-the-national-dpp.html [Last accessed: March 17, 2022].

[12] Mitchell NS, Polsky S, Catenacci VA, et al. Up to 7 years of sustained weight loss for weight-loss program completers. Am J Prev Med 2015;49(2):248–258; doi: 10.1016/j.amepre.2015.02.011 26033350 PMC4811034

[13] Jolly K, Lewis A, Beach J, et al. Comparison of range of commercial or primary care led weight reduction programmes with minimal intervention control for weight loss in obesity: Lighten Up randomised controlled trial. BMJ 2011;343(2):d6500; doi: 10.1136/bmj.d6500 22053315 PMC3208022

[14] Jebb SA, Ahern AL, Olson AD, et al. Primary care referral to a commercial provider for weight loss treatment versus standard care: A randomised controlled trial. Lancet 2011;378(9801):1485–1492; doi: 10.1016/S0140-6736(11)61344-5 21906798 PMC3207352

[15] Heshka S, Anderson JW, Atkinson RL, et al. Weight loss with self-help compared with a structured commercial program. JAMA 2003;289(14):1792–1798; doi: 10.1001/jama.289.14.1792 12684357

[16] Foster‐Schubert KE, Alfano CM, Duggan CR, et al. Effect of diet and exercise, alone or combined, on weight and body composition in overweight‐to‐obese postmenopausal women. Obesity (Silver Spring) 2012;20(8):1628–1638; doi: 10.1038/oby.2011.76 21494229 PMC3406229

[17] Brunisholz KD, Kim J, Savitz LA, et al. A formative evaluation of a diabetes prevention program using the RE-AIM framework in a learning health care system, Utah, 2013–2015. Prev Chronic Dis 2017;14:E58; doi: 10.5888/pcd14.160556 28727546 PMC5524524

[18] Mattke S, Kapinos K, Caloyeras JP, et al. Workplace Wellness Programs: Services Offered, Participation, and Incentives. 2014. Available from: https://www.dol.gov/sites/dolgov/files/EBSA/researchers/analysis/health-and-welfare/workplace-wellness-programs-services-offered-participation-and-incentives-report.pdf [Last accessed: July 22, 2024]. 10.7249/rhq-05-02-07 PMC5158287 28083383

[19] Tsai AG, Wadden TA. Systematic review: An evaluation of major commercial weight loss programs in the United States. Ann Intern Med 2005;142(1):56–66; doi: 10.7326/0003-4819-142-1-200501040-00012 15630109

[20] Cannon MJ, Masalovich S, Ng BP, et al. Retention among participants in the national diabetes prevention program lifestyle change program, 2012–2017. Diabetes Care 2020;43(9):2042–2049; doi: 10.2337/dc19-2366 32616617 PMC11000538

[21] Murray J, Craigs CL, Hill KM, et al. A systematic review of patient reported factors associated with uptake and completion of cardiovascular lifestyle behaviour change. BMC Cardiovasc Disord 2012;12(1):120; doi: 10.1186/1471-2261-12-120 23216627 PMC3522009

[22] Jackson MC, Dai S, Skeete RA, et al. An examination of gender differences in the National Diabetes Prevention Program’s lifestyle change program. Diabetes Educ 2020;46(6):580–586; doi: 10.1177/0145721720964585 33063641 PMC7802597

[23] Monda V, Salerno M, Fiorenzo M, et al. Role of sex hormones in the control of vegetative and metabolic functions of middle-aged women. Front Physiol 2017;8:773; doi: 10.3389/fphys.2017.00773 29046646 PMC5632804

[24] Baucom KJW, Pershing ML, Dwenger KM, et al. Barriers and facilitators to enrollment and retention in the national diabetes prevention program: Perspectives of women and clinicians within a health system. Womens Health Rep (New Rochelle) 2021;2(1):133–141; doi: 10.1089/whr.2020.0102 34036296 PMC8139255

[25] Harris PA, Scott KW, Lebo L, et al. ResearchMatch: A national registry to recruit volunteers for clinical research. Acad Med 2012;87(1):66–73; doi: 10.1097/ACM.0b013e31823ab7d2 22104055 PMC3688834

[26] R Core Team. A Language and Environment for Statistical Computing. R Foundation for Statistical Computing [computer program]: Vienna, Austria; 2020.

[27] Centers for Disease Control and Prevention. Assessing Your Weight. 2020. Available from: https://www.cdc.gov/healthyweight/assessing/index.html [Last accessed: January 24, 2022].

[28] Centers for Disease Control and Prevention. How Much Physical Activity to Adults Need? 2020. Available from: https://www.cdc.gov/physicalactivity/basics/adults/index.htm [Last accessed: January 24, 2022].

[29] Verheijden MW, Jans MP, Hildebrandt VH, et al. Rates and determinants of repeated participation in a web-based behavior change program for healthy body weight and healthy lifestyle. J Med Internet Res 2007;9(1):e1; doi: 10.2196/jmir.9.1.e1 17478410 PMC1794672

[30] Alharbi M, Gallagher R, Neubeck L, et al. Exercise barriers and the relationship to self-efficacy for exercise over 12 months of a lifestyle-change program for people with heart disease and/or diabetes. Eur J Cardiovasc Nurs 2017;16(4):309–317; doi: 10.1177/1474515116666475 27562115

[31] Elgaddal N, Dramarow EA, Reuben C. Physical activity among adults aged 18 and over: United States, 2020. In: NCHS Data Brief. National Center for Health Statistics: Hyattsville, MD; 2022;443. Available from: https://www.cdc.gov/nchs/data/databriefs/db443.pdf. Accessed July 22, 2024. 36043905

[32] National Institute of Diabetes and Digestive and Kidney Diseases (NIKKD). Overweight & Obesity Statistics. 2018. Available from: https://www.niddk.nih.gov/health-information/health-statistics/overweight-obesity Published 2018. Updated September 1, 2021 [Last accessed: June 26, 2024].

