# Supplementary material for: Motivators and Barriers to Joining a Lifestyle Change Program for Disease Prevention
Source: Womens Health Rep (New Rochelle). 2024 Sep 6;5(1):624–31. doi: 10.1089/whr.2024.0093 (PMC11462425; doi:10.1089/whr.2024.0093)
Supplement: Supplementary Appendix S1 [file whr.2024.0093_Supplement.pdf]

Appendix 1: The survey instrument

<u>Feasibility Study on Lifestyle Change Programs</u>

1. How old are you? ______ (If not between 40-74 → **Please stop and return this form)**
2. Have you ever been diagnosed with breast cancer? ❑ No ❑ Yes→ **Please stop**
3. Have you been diagnosed with diabetes? ❑ No ❑ Yes→ **Please stop**

(If you have been diagnosed with pre-diabetes, mark No)

4. Are you currently pregnant? ❑ No ❑ Yes→ **Please stop**

Lifestyle change programs encourage people to be healthier, typically through healthy eating, weight loss, and/or physical activity. Many of these programs last for up to one year and include weekly or monthly meetings. Some programs are done in person, others are online.

The University of Utah is planning a **research study** designed to encourage women to join lifestyle change programs. These programs can help you lose weight and reduce your chance of being diagnosed with many different types of diseases. The study includes 3 in-person visits – one after a mammogram visit, one 6 months later, and one 12 months later. Each of these visits would happen at the University of Utah and you would receive $25 for each visit.

	Not at all interested				Very interested
5. How interested are you in joining this kind of study if there was **no cost to you** for the lifestyle program?	❑	❑	❑	❑	❑
6. How interested are you in joining this kind of study if you had to **pay for the lifestyle program**?	❑	❑	❑	❑	❑

7. If you would be willing to pay for a lifestyle program in this kind of study, how much would you be willing to pay?
   Please list an amount between $0 - $600: ______________________________

As a part of this study, how interested are you in joining each of the lifestyle change programs described below that focuses on healthy eating, weight loss, and/or physical activity? Please exclude cost from your answer.

	Not at all interested				Very interested
8. **Weight Watchers**. A program focused on weight loss.	❑	❑	❑	❑	❑

**9. Diabetes Prevention Program**. A program to help prevent type 2 diabetes through weight loss and physical activity.	❑	❑	❑	❑	❑
**10. TOPS**. A nonprofit network of weight-loss support groups and wellness education organizations.	❑	❑	❑	❑	❑
**11. Other(s)**, please specify: _______________________	❑	❑	❑	❑	❑

12. Are you aware that the Diabetes Prevention Program (DPP) and similar lifestyle programs decrease the risk for the following diseases in addition to diabetes?

Heart disease	❑ Yes	❑ No
Breast Cancer	❑ Yes	❑ No
Hypertension	❑ Yes	❑ No
Arthritis	❑ Yes	❑ No

Other(s), please specify:______________________________________

13. Mark the following disease(s) that would motivate you to join a program like the DPP.

❑ Heart disease
❑ Breast Cancer
❑ Hypertension
❑ Arthritis
❑ Other(s), please specify:______________________________________

14. Mark the barrier(s) for you to joining these kinds of programs.

❑ Cost
❑ Language
❑ Not at convenient locations
❑ Not at convenient times
❑ Too many in-person meetings
❑ Too few in-perosn meetings
❑ Other(s), please specify:______________________________________

15. What is your height? ____ feet ____ inches

16. What is your weight? ______ pounds

17. How many minutes of physical activity/exercise do you get in a typical week?

❑ None
❑ Less than 30
❑ 30-90
❑ 91-150
❑ More than 150

**Do you have a…**	**Yes, Type 1 Diabetes**	**Yes, Type 2**	**No**	**Unsure**

**18.** Sister or brother with diabetes?	❑	❑	❑	❑
**19.** Parent with diabetes?	❑	❑	❑	❑

20. What is your ethnicity?

- ❑ Hispanic/Latina
- ❑ Not Hispanic/Latina
- ❑ Other, please specify: _________________________

21. What is your race?

- ❑ American Indian and Alaska Native
- ❑ Asian
- ❑ Black or African American
- ❑ Native Hawaiian and Other Pacific Islander
- ❑ White or Caucasian
- ❑ Other, please specify: _________________________

22. What is your native language(s)?

- ❑ English
- ❑ Spanish
- ❑ Other, please specify: _________________________

Thank you for your time. Please return this survey to the person who gave it to you.
